# Sodium Thiosulfate Ameliorates Oxidative Stress and Preserves Renal Function in Hyperoxaluric Rats

**DOI:** 10.1371/journal.pone.0124881

**Published:** 2015-04-30

**Authors:** Rakesh K. Bijarnia, Matthias Bachtler, Prakash G. Chandak, Harry van Goor, Andreas Pasch

**Affiliations:** 1 Department of Nephrology, Hypertension and Clinical Pharmacology, University Hospital and University of Bern, Inselspital, Bern, Switzerland; 2 Department of Clinical Research, University Hospital and University of Bern, Inselspital, Bern, Switzerland; 3 Department of Pathology and Medical Biology, University of Groningen, University Medical Center Groningen, Groningen, The Netherlands; University of Louisville, UNITED STATES

## Abstract

**Background:**

Hyperoxaluria causes crystal deposition in the kidney, which leads to oxidative stress and to injury and damage of the renal epithelium. Sodium thiosulfate (STS, Na_2_S_2_O_3_) is an anti-oxidant, which has been used in human medicine for decades. The effect of STS on hyperoxaluria-induced renal damage is not known.

**Methods:**

Hyperoxaluria and renal injury were induced in healthy male Wistar rats by chronic exposure to ethylene glycol (EG, 0.75%) in the drinking water for 4 weeks. The treatment effects of STS, NaCl or Na_2_SO_4_ were compared. Furthermore, the effects of STS on oxalate-induced oxidative stress were investigated *in vitro* in renal LLC-PK1 cells.

**Results:**

Chronic EG exposure led to hyperoxaluria, oxidative stress, calcium oxalate crystalluria and crystal deposition in the kidneys. Whereas all tested compounds significantly reduced crystal load, only STS-treatment maintained tissue superoxide dismutase activity and urine 8-isoprostaglandin levels *in vivo* and preserved renal function. In *in vitro* studies, STS showed the ability to scavenge oxalate-induced ROS accumulation dose dependently, reduced cell-released hydrogen peroxide and preserved superoxide dismutase activity. As a mechanism explaining this finding, STS was able to directly inactivate hydrogen peroxide in cell-free experiments.

**Conclusions:**

STS is an antioxidant, which preserves renal function in a chronic EG rat model. Its therapeutic use in oxidative-stress induced renal-failure should be considered.

## Introduction

Oxalate is a metabolic end product, which is excreted with the urine. In case of excessive nutritional intake or pathological overproduction (e.g. due to primary hyperoxaluria, enteric hyperoxaluria, EG toxicity or excessive vitamin C ingestion), calcium oxalate crystals precipitate throughout the body. The kidneys are most vulnerable to these crystals, which lead to nephrolithiasis, nephrocalcinosis and renal failure [1]. High oxalate concentrations cause oxidative stress, antioxidant depletion and injury to the renal epithelium [2, 3]. Oxidants in hyperoxaluria appear to be mainly mitochondria-derived [4] and therefore impair the intracellular antioxidant defense systems including the activity of enzymes like superoxide dismutase (SOD) and catalase (CAT) [3, 5].

Sodium thiosulfate (Na_2_S_2_O_3_, STS) is a sulfur salt, which has been used since decades in human medicine for the treatment of cyanide intoxications. Furthermore, various clinical case reports indicate that STS exhibits calcification-preventing properties [6, 7] and reduces calcium phosphate mineralization in humans suffering from calciphylaxis [8], in adenine-treated uremic rats [9] and in genetically hypercalciuric rats [10]. These anticalcifying properties of STS are potentially related to its antioxidant effects [11]. Despite this ability to prevent calcification, LaGrange and colleagues [12] reported that STS had no significant effect on crystalluria and calcium oxalate precipitation in a rat model of acute EG and NH_4_Cl-induced calcium oxalate nephropathy. The reason for not observing a beneficial effect of STS in this model has not been elucidated but may have been due to the acute, severe and irreversible kidney-damaging nature of this model.

We, therefore, hypothesized that STS might have beneficial effects in a chronic and modest model of oxalate-induced oxidative stress and kidney damage [3, 13].

To test this hypothesis, we exposed male Wistar rats to 0.75% EG in the drinking water for 28 days. During these 4 weeks, the rats received intraperitoneal (i.p.) injections of either STS, NaCl (SC) or Na_2_SO_4_ (SS). Furthermore, the effect of STS on oxidative stress was investigated using an *in vitro* system of proximal tubular LLC-PK1 cells.

## Material and Methods

### Animal study

The animals were purchased from Charles River, Germany and the experiments were performed with all permissions required by the Swiss authorities (Sekretariat Tierversuche Herrengasse 1, 3011, Bern, Switzerland, Permission number BE87/11). Male Wistar rats (n = 40) weighing between 100 and 150 g were randomly divided into five groups of eight animals each. Group I (Control) received normal rat chow and drinking water and no additional treatment. Groups II through V received ethylene glycol (EG, Fluka) at a dose of 0.75% (v/v) with the drinking water. Group III (EG + STS) was in addition treated with sodium thiosulfate (Dr. Franz Köhler Chemie GmbH, Bensheim, Germany), at a dosage of 0.4 g/kg body weight thrice a week. Group IV (EG + SC) and Group V (EG + SS) received i.p. injections of sodium chloride and sodium sulfate respectively at doses of 0.4 g/kg body weight thrice a week. All treatments were continued for 28 days. 24-hour urine was collected in metabolic cages at week 0, 2 and 4. On the day of sacrifice, blood was drawn and both kidneys were removed and fixed in buffered formaldehyde. Some of the tissue was frozen in liquid nitrogen and stored at -80°C until further use. The animals were killed by an overdose intraperitoneal injection of sodium pentobarbitals.

### Urine and serum biochemistry

Calcium and creatinine in urine and serum were measured using calcium and creatinine assay kits (QuantiChrome, DICA-500 and DICT-500). Urinary oxalate was measured by HPLC in the clinical chemistry facility at University Hospital Bern. The OxiSelect 8-iso-Prostaglandin F2a ELISA Kit (Cell Biolabs, INC., STA 337) was used to measure 8-IP in the urine.

### Processing of Kidney Tissue

For observing crystals and other tissue histological changes, the sections were stained with hematoxylin and eosin. Crystals were counted using light microscopy. For this purpose, using the 10x magnification, 12 fields were randomly selected which typically covered almost the whole area of a kidney cross section. For α-SMA immunostaining, sections were stained with primary antibodies for macrophages (mouse anti-§CD68 ED1, MCA341R AbD, 1:750, Serotec Ltd, Oxford, UK) and α-Smooth Muscle Actin (mouse anti- α SMA, clone 1A4 A2547, 1:10.000, Sigma, Zwijndrecht, the Netherlands). Kidney sections were scanned using an Aperio Scanscope GS (Aperio Technologies, Vista, CA, USA). The extent of pre-fibrotic changes (α-SMA, excluding vessels) was quantified using computer-assisted analysis with Aperio Imagescope (Version 9.1, Aperio, Vista, CA, USA). For α-SMA and ED1 the ratio between the relative cortical staining and the total cortical surface area was used. All computerized measurements were performed in a blinded manner [14]. For osteopontin (OPN) immunostaining, the sections were stained with primary antibodies for osteopontin (mouse anti-OPN, clone MPIIIB10, 1:300, Developmental Hybridoma Studies Institute, Iowa City, IA, USA), The deparaffinized sections were washed with phosphate buffered saline (PBS, pH 7.4) and overnight antigen retrieval process was performed in 0.1 M Tris/HCl buffer, pH 9.0, at 80°C. The sections were again washed with PBS, blocked with endogenous peroxidase by treating with 0.3% H_2_O_2_ in PBS for 30 minutes and incubated with primary antibodies for 60 minutes at room temperature. Binding was detected using sequential incubation with primary antibodies associated peroxidase-labeled secondary and tertiary antibodies (Dakopatts, Glostrup, Denmark) for 30 minutes. All antibodies were diluted with PBS containing 1% Bovine Serum Albumin (BSA) and 1% normal rat serum was added to the secondary and tertiary antibody dilutions. Peroxidase activity was developed using 3,3’-diaminobenzidine tetrachloride (DAB) for 10 minutes containing 0.03% H_2_O_2_. Counterstaining was performed using Mayer’s hematoxylin. Appropriate isotype and PBS controls were consistently negative.

To measure the activities of the enzymes superoxide dismutase (SOD) and catalase (CAT) [15], renal tissue was immediately put into PBS-containing 0.5 mg/ml butylated hydroxytoluene (BHT) to avoid oxidation and was then homogenized on dry ice. The activity of SOD and CAT were measured using kits from Sigma (cat.no. 19160) for SOD, and from BioVision (cat.no. K-773) for CAT, respectively.

### Cell culture

The porcine proximal tubular kidney cell line LLC-PK1 was obtained from the American Type Culture Collection (CL-101). Cells were maintained in 75 cm^2^ Falcon T-flasks in DMEM (pH 7.4) containing 10% fetal bovine serum, minimal essential medium (1%), sodium pyruvate (1 mM), streptomycin (0.1 mg/ml) and penicillin (100 IU/ml) at 37°C and 5% CO_2_. For experiments, confluent monolayers were used, and all experiments were carried out in PBS or in serum- and pyruvate-free DMEM media. To avoid confounding, potential interactions of STS with the kits and chemicals used were carefully ruled out.

### STS uptake into LLC-PK1 cells

For this experiment, the LLC-PK1 cells were kept in 100 μM STS for different time periods and the concentration of STS in the supernatant was measured by HPLC [16].

### STS toxicity studies

Confluent LLC-PK1 cells, cultured in 48 well plates for 24 hrs were used for toxicity studies. STS (20 mM) was added to these cells for 6 and 24 hrs in serum free media. After the treatment, cells were fixed (4% formaldehyde for 1 hr) and a protein staining dye-sulforhodamine (0.057% sulforhodamine in 1% acetic acid) was used to determine their viability, by exposing it to cells for 30 min. Before measuring the absorbance at 570 nm, cells were washed with 1% acetic acid (4 times), air dried, dissolved in 10 mM TRIS base (pH 10.5) and were put on a shaker for 5 min for complete dissolution of dye. The percentage change in absorbance with respect to control was calculated for all samples.

### Impact of STS on Reactive Oxygen Species (ROS)

To measure SOD activity, LLC-PK1 cells were cultured in 6-well plates (1x10^6^ cells per well in 1ml DMEM) for 24 hrs. The 70% confluent wells were washed with PBS gently and pre-treated with STS, SC or SS (1 mM each) for 12 hrs. Oxalate (2 mM) was dissolved in serum-, phenol red- and pyruvate-free media, mixed with STS, SC or SS (1 mM) and subsequently added to the cells for 24 hrs. Phenol red-free media was used to avoid interferences with the colorimetric SOD assay kit (Sigma, 19160).

ROS were measured using 5-(and-6)-carboxy-2’,7’-difluorodihydrofluorescein diacetate (Carboxy-H_2_DFFDA). LLC-PK1 cells were cultured for 24 hrs in 96-well plates (10^3^ cells/ml, 200 μl DMEM media). Then, the cells were pre-treated with STS (1 mM), SC (1 mM) or SS (1 mM) for 12 hrs. Following this pre-treatment, oxalate (1 mM), STS (or SC or SS) and Carboxy-H_2_DFFDA dye (10 μM) were added simultaneously to the cells in PBS. After keeping the plate in the dark for different time intervals, fluorescence was measured using a Fluoroscan spectrophotometer at an excitation wavelength of 485 nm and an emission wavelength of 538 nm in one-hour intervals. Eight replicates were used for each condition.

As an alternative approach, hydrogen peroxide content was determined following oxalate exposure in the presence and absence of treatments. LLC-PK1 cells were cultured in 24-well plates (5x10^4^ cells per well in 500μl DMEM) for 24 hrs followed by pre-treatment with STS, SC or SS (1 mM each) for 12 hrs. Oxalate (1 mM) was dissolved in serum-, phenol red- and pyruvate-free media, mixed with STS, SC or SS (1 mM) and subsequently added to the cells for 72 hrs. Phenol red-free media was used to avoid interferences with the colorimetric hydrogen peroxide assay. Following the treatment period, the cell supernatant was centrifuged at 9.500xg and hydrogen peroxide was determined using a commercially available kit (Biovision, K-265).

In addition to above experiment, the direct interactions of STS, SS or SC with hydrogen peroxide were tested in a cell-free system. To this end, STS, SC or SS (1–7 mM each) were dissolved in 10 ml PBS (10 mM) containing 50 mM H_2_O_2_. After 3 hrs at room temperature, H_2_O_2_ concentration changes were measured.

### Statistical analysis

Differences between animal groups were analyzed and all graphs plotted using the GraphPad software (GraphPad Software Inc., La Jolla, CA, USA). All results, including the graphs in the figures, are given as means ± s.d. and statistical analysis was done by Students *t*-test, Mann Whitney analysis and one-way analysis of variance (ANOVA) with Bonferroni’s multiple comparison tests as appropriate.

## Results

### Effects of STS in an *in vivo* model of oxidative stress

**Fig 1:**
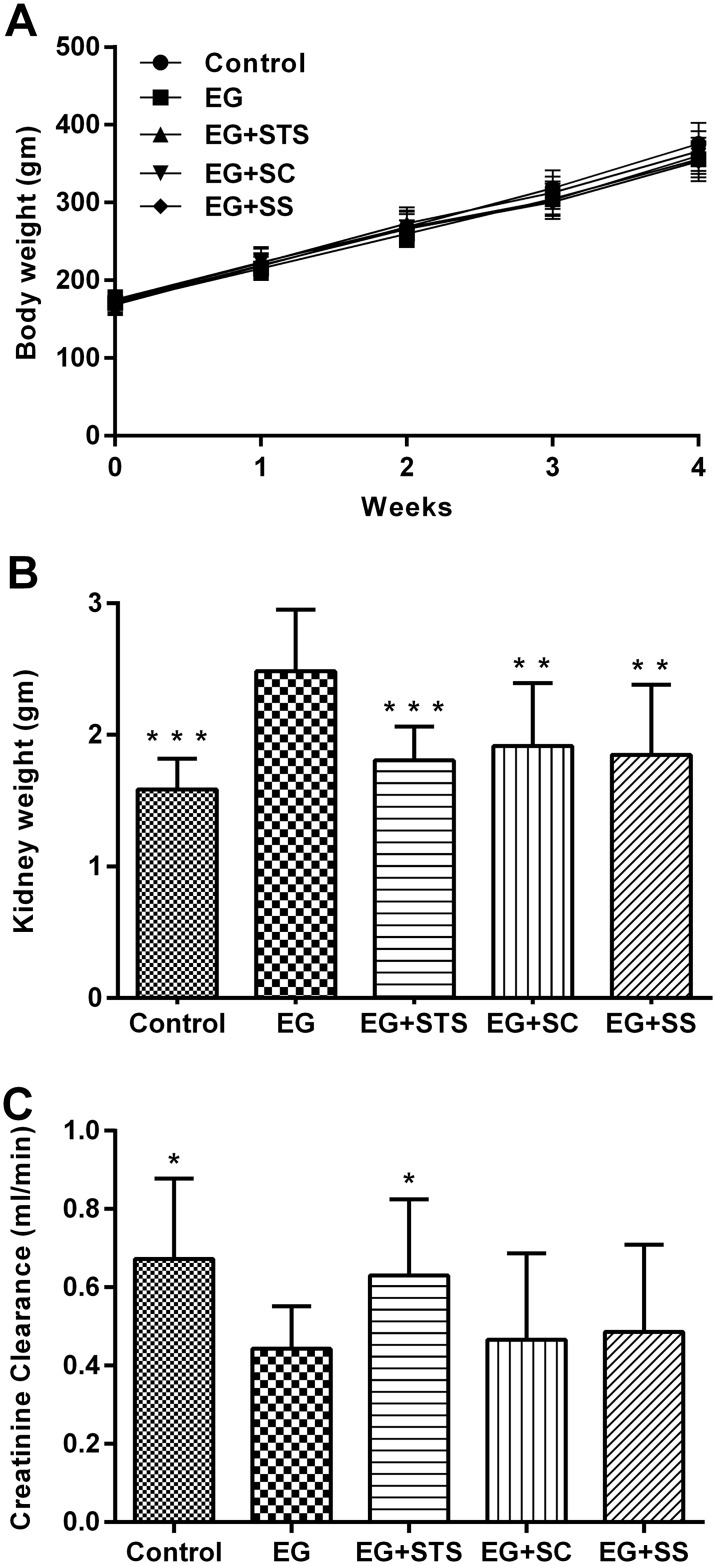
Treatment effects on body weight, kidney weight and renal function. (A) Initial body weight and weight gain were similar in all animal groups irrespective of the treatment modality. (B) Kidney weight was increased in the EG group, but near-to-normal in the EG+STS, EG+SC and EG+SS groups. (C) Creatinine clearance derived from 24-hour urine samples collected after 4 weeks shows preserved renal function in STS-treated animals. The significance levels are with reference to the EG group. Data are presented as means ± SD from 7–8 animals per group *p< 0.05, **p < 0.01, ***p < 0.001.

Using a chronic EG-induced hyperoxaluria rat model of oxidative stress, the effects of sodium thiosulfate (Na_2_S_2_O_3_, STS), sodium chloride (NaCl, SC) and sodium sulfate (Na_2_SO_4_, SS) on renal function, renal histology and oxidative stress were studied. To this end, urine and blood were collected at 0, 2 and 4 weeks of treatment with 0.75% EG in the drinking water and the rats sacrificed and renal histology analyzed at 4 weeks. Throughout the experiment, all animals behaved normally and looked healthy except for one animal from the SS group, which died without a detectable reason. Body weight was comparable in all groups (Fig 1A).

As expected, polyuria was induced by the EG treatment. This was somewhat attenuated in the treatment groups (Table 1). Kidney weight was significantly increased in the hyperoxaluric rats when compared to control animals (p < 0.01, Fig 1B). STS prevented the increase of kidney weight (p < 0.001), an effect that was also found as a trend in the sodium chloride (SC) and sodium sulfate (SS) treated groups. Interestingly, serum creatinine and renal creatinine clearance of STS-treated animals were comparable to control animals. This protection of renal function was found in the STS-treated animals only (Fig 1C).

**Table 1:** Urine and serum biochemistry.

		Control	EG	EG+STS	EG+SC	EG+SS
Urine	Volume, ml/d T_0_	7.5 ± 2.0	7.2 ± 3.0	7.2 ± 2.6	8.6 ± 1.8	9.8 ± 2.8
	Volume, ml/d T_2_	13.2 ± 3.6	25.6 ± 11.8	21.1 ± 6.9	19.4 ± 10.0	19.1 ± 9.6
	Volume, ml/d T_4_	17.2 ± 6.7	31.0 ± 5.9	26.4 ± 6.1	22.7 ± 9.9	23.4 ± 6.6
	Oxalate (μmol/d, T_4_)	12.33 ± 1.64*	93.72 ± 40.24	76.53 ± 37.13	53.63 ± 17.39	58.50 ± 30.98
	Calcium (mg/d, T_4_)	3.55 ± 1.39*	5.33 ± 0.96	4.98 ± 0.98	3.96 ± 1.42*	4.45 ± 1.06
	Creatinine (mmol/d, T_4_)	0.048 ± 0.01*	0.037 ± 0.00	0.044 ± 0.00*	0.038 ± 0.01	0.033 ± 0.00
Serum	Calcium (mg/dl, T_4_)	9.74 ± 1.19	9.72 ± 0.56	9.85 ± 1.25	9.00 ± 1.17	9.32 ± 1.24
	Creatinine (μM, T_4_)	50.73 ± 8.4*	63.03± 11.0	50.84 ± 8.2*	61.50± 14.8	57.23 ± 23
CrCl (ml/min, T_4_)		0.67 ± 0.20*	0.44 ± 0.10	0.63 ± 0.19*	0.46 ± 0.22	0.48 ± 0.22

Data are means ± SD from 7–8 animals per group. Significance levels are with reference to the EG group.

*p< 0.05 is statistically significant. T_0_ = week 0, T_2_ = week 2, T_4_ = week 4.

**Fig 2:**
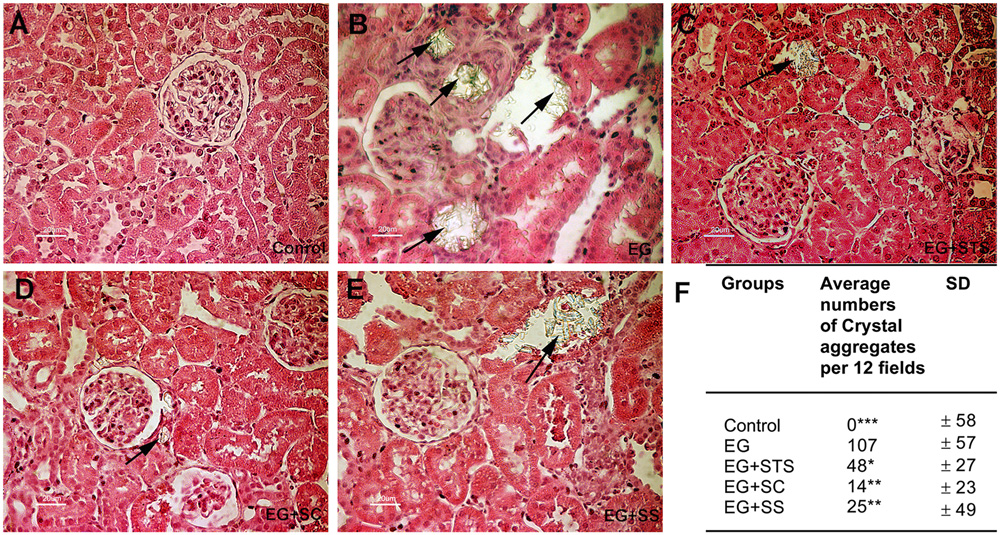
EG-induced changes of kidney histology. Representative hematoxylin- and eosin-stained kidney sections obtained from (A) control, (B) EG-exposed, (C) EG+STS-exposed, (D) EG+SC-exposed, (E) EG+SS-exposed animal groups. Arrows indicate calcium oxalate crystal deposits. (F) Results of light microscopic quantification of crystal aggregates. The data are means ± SD from 7–8 animals per group. The significance levels are with reference to the EG group. *p< 0.05, **p < 0.01, ***p < 0.001.

**Fig 3:**
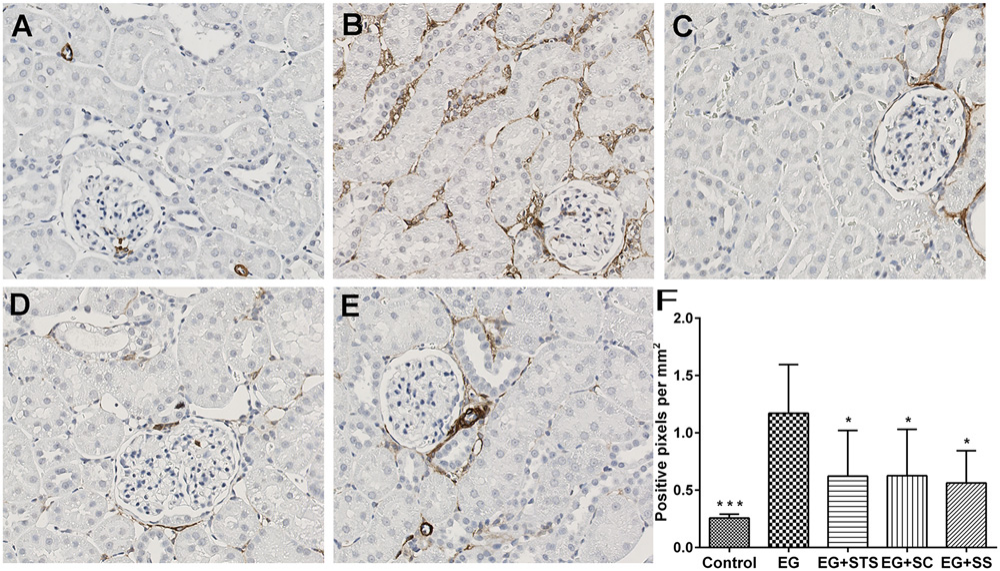
α-SMA stain of kidney tissue. The groups are (A) control, (B) EG-exposed, (C) EG+STS-exposed, (D) EG+SC-exposed, (E) EG+SS-exposed animals. The α -SMA stain was markedly increased in EG-exposed animals but decreased in all treatment groups. The significance levels are with reference to the EG group. The data are means ± SD from 7–8 animals per group. *p< 0.05, **p < 0.01, ***p < 0.001.

**Fig 4:**
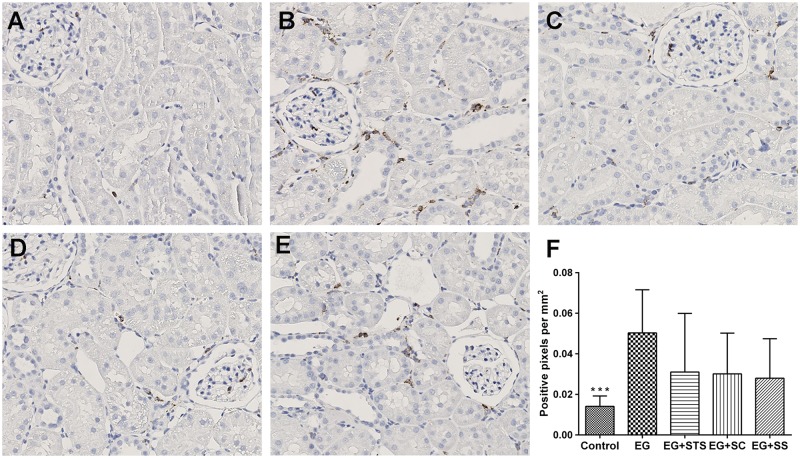
ED-1 stain of kidney tissue. The groups are (A) control, (B) EG-exposed, (C) EG+STS-exposed, (D) EG+SC-exposed, (E) EG+SS-exposed animal groups. The ED-1 stain was markedly increased in EG-exposed animals but decreased in all treatment groups. The significance levels are with reference to the EG group. The data are means ± SD from 7–8 animals per group. ***p < 0.001.

**Fig 5:**
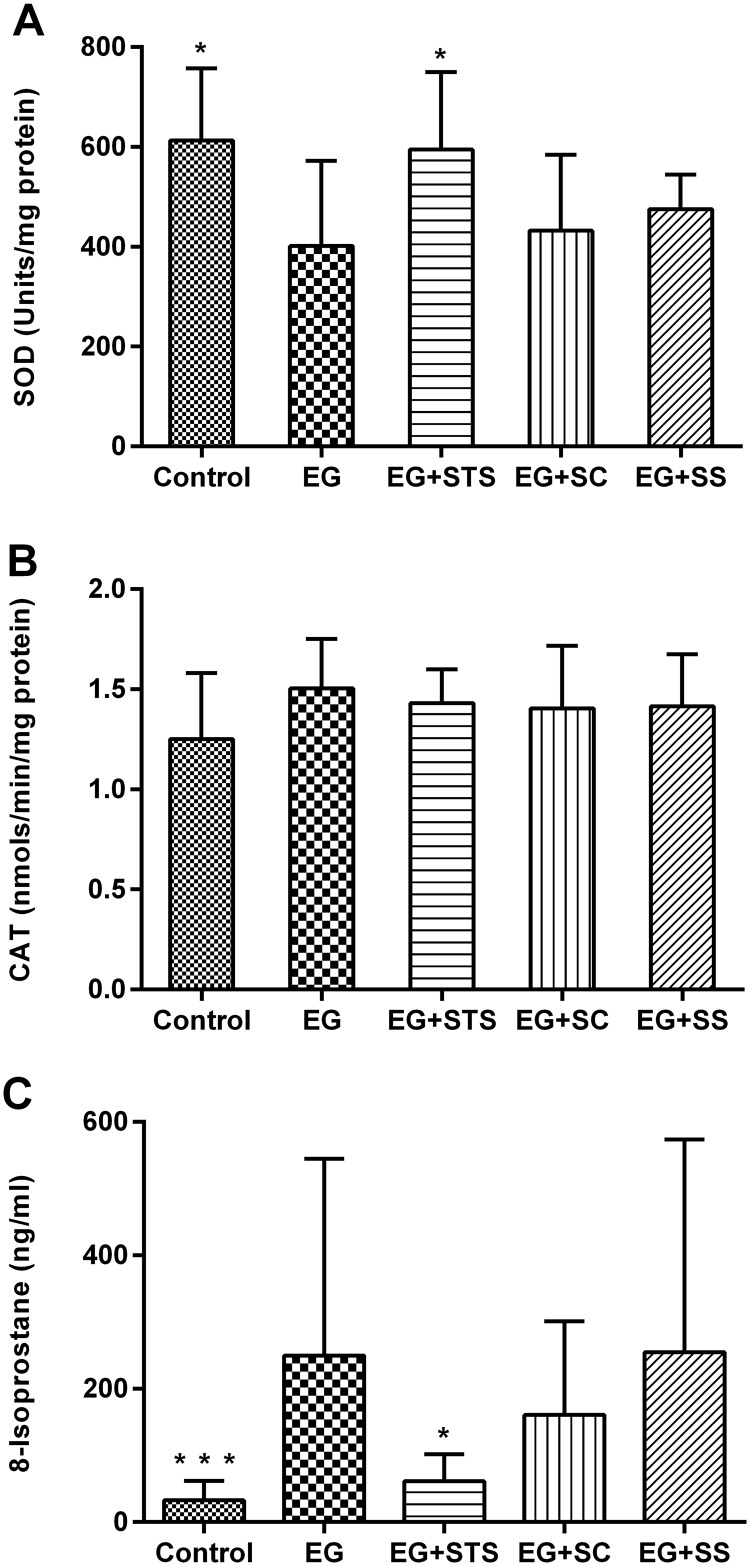
Oxidant-antioxidant status. The activities of the two antioxidant enzymes (A) Superoxide dismutase (SOD), and (B) Catalase (CAT) were determined in renal tissue at time point 4 weeks in the control, EG, EG+STS, EG+SC and EG+SS animal group. SOD activity was preserved with STS treatment; (C) Urinary 8-Isoprostaglandin levels were elevated in the EG-exposed animals and maintained in the normal range only in STS-treated animals. The changes and significance levels are with reference to the EG group. The data are means ± SD from 7–8 animals per group. *p< 0.05, **p < 0.01, ***p < 0.001.

The histo-morphological analysis of kidney tissue from different groups revealed the presence of extensive calcium oxalate crystal aggregates in the tubules of EG-treated rats, (Fig 2A–2E). Quantification of these aggregates revealed that all three treatment modalities ameliorated the crystal load in the order SC > SS > STS when compared to EG (Fig 2F). The α-SMA staining score, an indicator of interstitial fibrosis, was very high in EG-treated animals (Fig 3A–3F) but was reduced significantly by all three treatments. Similarly, the macrophage marker, ED-1 was highly expressed in the EG-exposed kidneys and reduced in the treated kidneys (Fig 4A–4F). Given that STS had a comparatively weak effect on the prevention of crystal deposition as well as smooth muscle actin and macrophages stains, we reasoned, that the preservation of renal function found in the STS group (Fig 1C) had not been due to the prevention of calcium oxalate crystal deposition in the kidneys. Although the crystal load was less with the treatments, however, the oxalate excretion remained elevated significantly among all treatments (Table 1) compared to animals treated with EG alone. To investigate the oxidative stress induced by EG exposure, we determined the activity of the antioxidant enzymes SOD and CAT [14] in renal tissue and the level of 8-IP in the urine (Fig 5). While SOD was significantly reduced (p<0.05) in EG-exposed animals, animals treated with STS showed normal SOD activity (Fig 5A). In contrast, both control groups exhibited clearly reduced SOD activity. Upon analyzing CAT activity, no significant changes were found in all five treatment and control groups (Fig 5B). The oxidative stress marker, 8-isoprostaglandin was enhanced in the urine significantly upon EG-exposure and STS ameliorated this increase significantly (Fig 5C). Osteopontin (OPN), an inhibitor of calcium oxalate crystal formation was found to be expressed in the renal tissue upon EG exposure as indicated by the OPN staining score. No significant differences in the OPN expression were however observed among the treatment groups (S2 Fig).

As all three beneficial effects of STS, the preservation of creatinine clearance, SOD activity and 8-IP levels closely paralleled each other, we chose to further investigate the antioxidant properties of STS *in vitro*.

### Effects of STS in an *in vitro* model of oxidative stress

**Fig 6:**
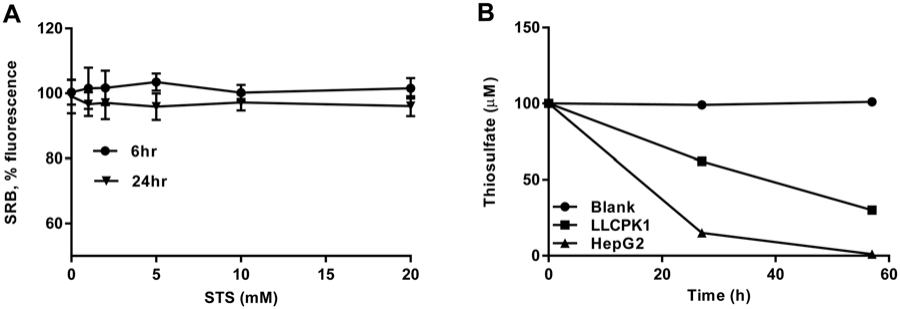
STS is non-toxic and can be incorporated by the LLC-PK1 cells. (A) *In vitro* toxicity studies with LLC-PK1 cells showed no significant changes in cell viability even at 20 mM STS for a period of 24 hrs in serum and pyruvate free DMEM media. (B) STS is decreasing in the supernatant of LLC-PK1 cells as detected by HPLC (HepG2 used as positive control and blank wells as negative control).

**Fig 7:**
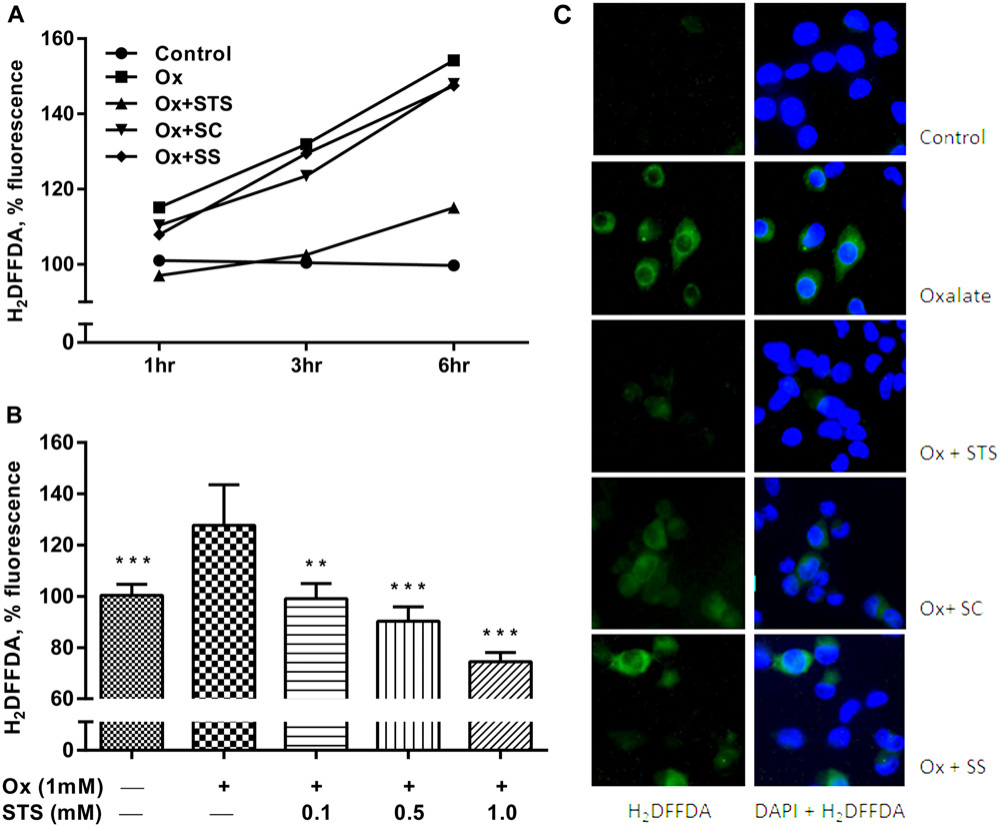
STS reduces intracellular oxidative stress. Oxidative stress was induced by exposure of proximal tubular LLC-PK1 cells towards 1 mM oxalate, and detected using the fluorescence dye H_2_DFFDA. (A) Fluorescence changes in untreated LLC-PK1 cells (Control), oxalate (Ox)-exposed LLC-PK1 cells (1 mM), and Ox+STS, Ox+SC and Ox+SS-exposed LLC-PK1 cells. STS largely protects LLC-PK1 cells against oxalate-induced oxidative stress. (B) Dose-dependent protection by STS against oxalate-induced ROS in LLC-PK1 cells. Data were analyzed by one-way analysis of variance (ANOVA) with Bonferroni’s multiple comparison. Asterisks (*) indicate statistically significant differences (*p <* 0.05) with respect to oxalate-exposed cells. (C) H_2_DFFDA- and DAPI-fluorescence imaging of LLC-PK1 cells. The absence of green fluorescence in oxalate-exposed, STS-treated LLC-PK1 cells indicates the quenching of intracellular oxidative stress by STS. Control = 100% in (A) and (B). Significance levels are with reference to oxalate (Ox) group. The data are means ± SD from 8 values per group. *p< 0.05, **p < 0.01, ***p < 0.001.

**Fig 8:**
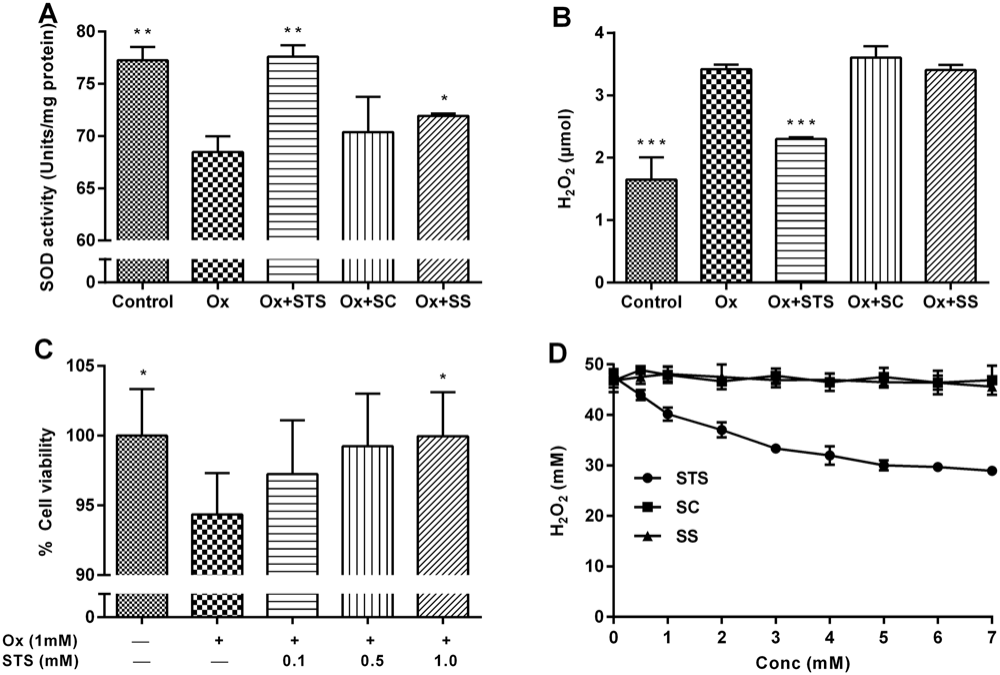
Intra- and extracellular quenching of H_2_O_2_ by STS. (A) SOD activity was rescued by STS treatment upon 24 hours of oxalate-exposure to LLC-PK1 cells. (B) Oxalate-exposure of LLC-PK1 cells leads to the intracellular generation and accumulation of H_2_O_2_. While STS-treatment reduced the amount of H_2_O_2_ released from the cells after 72 hours, SC- and SS-treatment did not. (C) 72 hours of oxalate-exposure of LLC-PK1 cells leads to a significant decrease in cell viability (measured by sulforhodamine), which was maintained by STS in a dose dependent manner. (D) STS directly quenches H_2_O_2_ in aqueous solution, providing evidence for a direct interaction between STS and the non-radical oxidant H_2_O_2_. SC and SS in contrast did not lead to the consumption of H_2_O_2_. The changes and significance levels are with reference to the Ox group. The data are means ± SD from 8 values per group. *p< 0.05, **p < 0.01, ***p < 0.001.

To investigate the antioxidant effect of STS with kidney cells *in vitro*, the proximal tubular cell line LLC-PK1 was chosen because (i) it possesses the capacity to metabolize STS (Fig 6B) and (ii) proximal tubular cells are vulnerable to ischemia and oxidative stress [17]. Potential STS toxicity was ruled out by exposing LLC-PK1 cells to 1 to 20 mM STS for 24 hrs and determining cell viability using sulforhodamine (Fig 6A). For the investigation of intracellular reactive oxygen species, H_2_DFFDA was used. This non-fluorescent compound becomes fluorescent upon reacting with reactive oxygen species (ROS) within cells. To avoid cell culture media-induced auto-fluorescence, all experiments were carried out in PBS (S1A Fig). When LLC-PK1 cells were exposed to free oxalate (1 mM), increasing amounts of ROS were generated over 6 hrs (Fig 7A). Simultaneous exposure to STS (1 mM) dramatically reduced ROS while SC and SS did not (Fig 7A). Fig 7B shows the dose-dependent ROS-quenching effect of STS over 3 hours. Fluorescence microscope imaging corroborated this finding (Fig 7C). Exposure of LLC-PK1 cells to oxalate (2 mM) for 24 hrs caused a significant depletion of SOD, which was maintained by STS (1mM) (Fig 8A). Furthermore, the exposure of LLC-PK1 cells to oxalate for 72 hrs led to the release of significant amounts of H_2_O_2_ into the supernatant (Fig 8B). This was not the case when the cells were exposed to STS, indicating that the presence of STS had led to a reduction of H_2_O_2_ in the supernatant (Fig 8B). The 72-hour oxalate exposure to LLC-PK1 caused a significant reduction in cell survival, which was maintained by STS dose-dependently (Fig 8C). The direct interaction of STS with H_2_O_2_ was confirmed in a cell-free experiment (Fig 8D). In contrast to STS, SC or SS did not affect H_2_O_2_ concentrations in this experiment. Taken together, these results indicate that STS is an antioxidant with the capacity to directly quench H_2_O_2_, both inside and outside of cells.

## Discussion

Chronic EG exposure leads to hyperoxaluria, renal crystal deposition, oxidative stress and impaired kidney function [18–22]. Given the increasing use of STS in humans [23–25] and its reported beneficial effects in calcifying/crystallizing conditions [11], we aimed to investigate the effect of STS on EG-induced crystal burden and renal dysfunction.

In our study, STS significantly reduced EG-induced crystal load in the renal tissue, an effect, which was paralleled by the reduction of the fibrosis marker α -SMA, the macrophage marker ED-1 and urinary oxalate excretion. Indicating a non-specific mechanism, these effects were not only observed in the STS-treated group, but also in the control animal groups treated with SC or SS. The uniform increase of osteopontin expression encountered in all EG-groups indicates that this crystal adhesive protein does not play a mechanistic role in this regard. A “washout” of the crystals or a simple dilution of urine preventing crystal formation also appears unlikely to explain this finding given the comparable urine volume of all EG-treated groups. More likely, compatible changes of the ion activity products induced in the treatment and control groups may explain this finding [26]. Of note, a crystal reduction was not found in a more acute and aggressive EG-model where EG along with ammonium chloride was used to induce crystaluria [12] and potentially overruled the beneficial effects observed in our study.

Despite the comparable effects of all treatments on crystal load and tissue changes in our study, creatinine clearance [27] was preserved in the STS-treated animal group only, an effect potentially due to the proposed antioxidant effects of STS [11]. STS is rapidly excreted by the kidney [16] and partially exchanged and metabolized by proximal tubular cells [28].

Oxalate and calcium oxalate crystals cause the generation of superoxide radicals [29] mainly via PKC-regulated NADPH oxidase [30, 31] and xanthine oxidase (XO) [32] [33] pathways in the mitochondria [34]. Free radical oxidants are short lived and their dismutation to H_2_O_2_, which represents the major oxidant burden, occurs at a very rapid rate [35]. Concurrently, the pre-existing antioxident enzymes clear H_2_O_2_, but if the oxidant burden stays elevated, H_2_O_2_ accumulates, diffuses across membranes and at high concentrations damages lipids, proteins and DNA. Besides the generation of oxidants, the natural renal antioxidant system is impaired by oxalate and calcium oxalate crystals. Specifically, the cytoplasmic Cu/Zn SOD is sensitive to H_2_O_2_, which leads to a loss of the conformation of SOD and of the coordination bond between Cu^2+^/Zn^2+^ions [36].

Accordingly, in our study oxalate exposure led to a pronounced intracellular increase in ROS generation detectable by Carboxy-H_2_DFFDA fluorescent dye, and a release of H_2_O_2_ into the supernatant of LLC-PK1 cells. STS, but not SC or SS reduced H_2_O_2_ presumably according to the chemical reaction, 4H_2_O_2_ + S_2_O_3_^2-^ → 2SO_4_^2-^ + 2H^+^ + 3H_2_O [37], which is supported by own experiments showing the production of H^+^ upon mixing of H_2_O_2_ with STS (data not given). Here, STS directly reduced intracellular H_2_O_2_ in a dose-dependent manner and also showed reductive capacity towards a ferricyanide reducing antioxidant assay (S1B Fig). Furthermore, we found SOD activity to be decreased in EG-treated animal and to be restored by STS, a result corroborated in *in vitro* experiments. Furthermore, the oxidative stress marker urinary 8-Isoprostaglandin was elevated in the EG-exposed animals whereas it remained in the normal range in the urine of STS-treated animals. In contrast, no change in the activity of the natural antioxidant peroxide enzyme catalase [15] was found. This is in line with previous studies, which demonstrated a transient increase of CAT activity as an adaptive response in the early stages of hyperoxaluria but not after 42 days of chronic EG exposure [38, 39].

The lack of an increase in CAT activity might contribute to chronically elevated H_2_O_2_ tissue levels and explain the positive effects of H_2_O_2_-quenching compounds on the preservation of renal dysfunction [40].

As oxalate even at physiological concentrations and in the absence of calcium oxalate crystals causes significant oxidative renal cell injury [2], antioxidants like Vitamin E [13], polyphenol-rich plant extracts, N-acetylcycteine [38], taurine [41] and glutathione appear to be of use to protect against oxidative-stress-related disease conditions [15].

Interestingly, vascular media calcification has been identified as such a condition where H_2_O_2_ leads to the initiation of the osteogenic differentiation of vascular smooth muscle cells and thereby triggers vascular calcification [42]. STS prevents this type of vascular calcifications in uremic rats [9], and recently endogenous urinary thiosulfate excretion was demonstrated to be associated with a favorable cardiovascular risk profile and a survival benefit of renal transplant recipients [43].

In conclusion, our study demonstrates that STS preserves renal function in a chronic model of EG-induced hyperoxaluria. Our data indicate that this protective effect was independent of crystal load and at least in part due to the anti-oxidative properties of STS. Therapeutic application of STS may be of use in hyperoxaluria and other conditions characterized by the generation of the non-radical oxidant H_2_O_2_.

## Supporting Information

S1 Fig **(A) Analysis of auto-fluorescence of H2DFFDA in DMEM and PBS**. Fluorescence increases in serum free DMEM (media) and no change in H_2_DFFDA fluorescence was observed in PBS. **(B) The ferric cyanide (Fe3+) reducing antioxidant power (FRAP) assay of STS**. FRAP was performed to assess reducing capability. In case of STS and positive control N-acetyl cysteine (NAC), absorbance increased steadily with increasing concentrations whereas SS & SC did not show any change. The results demonstrate the electron donating properties of STS for neutralizing free radicals by forming stable products. The data are means ± SD from 8 values per group.
(TIF)

S2 Fig Quantification of osteopontin expression. The crystal adhesive protein, osteopontin (OPN) was assessed in the renal tissue sections. The OPN staining significantly increased in EG-exposed animals and remained elevated among all treatment groups indicating no impact of these molecules on OPN expression.
(TIF)
