# Supplementary figures and images for: Sodium Thiosulfate Ameliorates Oxidative Stress and Preserves Renal Function in Hyperoxaluric Rats
Source: PLoS One. 2015 Apr 30;10(4):e0124881. doi: 10.1371/journal.pone.0124881 (PMC4415920; doi:10.1371/journal.pone.0124881)

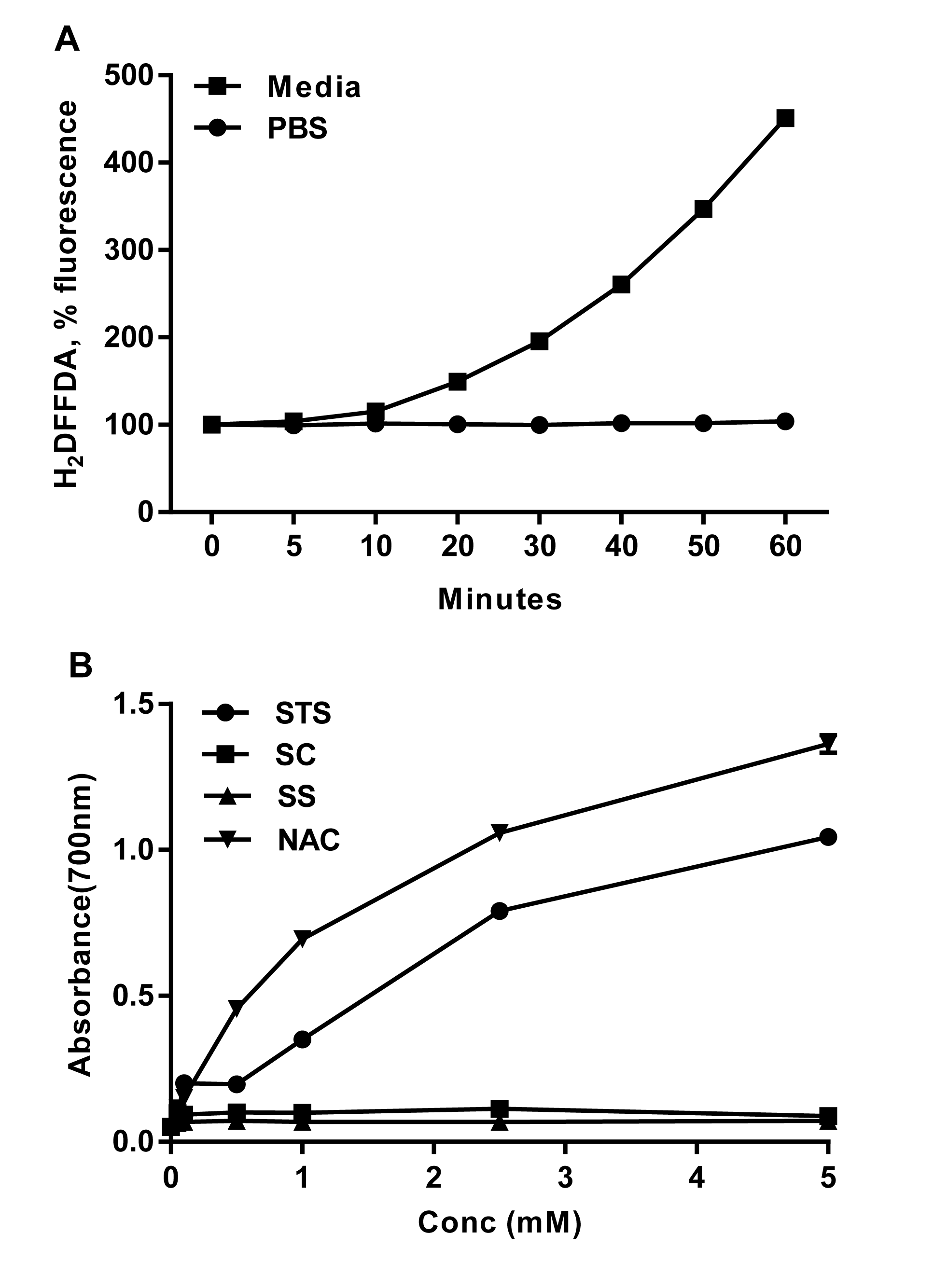

Supplement: S1 Fig — (A) Analysis of auto-fluorescence of H2DFFDA in DMEM and PBS. Fluorescence increases in serum free DMEM (media) and no change in H2DFFDA fluorescence was observed in PBS. (B) The ferric cyanide (Fe3+) reducing antioxidant power (FRAP) assay of STS. FRAP was performed to assess reducing capability. In case of STS and positive control N-acetyl cysteine (NAC), absorbance increased steadily with increasing concentrations whereas SS & SC did not show any change. The results demonstrate the electron donating properties of STS for neutralizing free radicals by forming stable products. The data are means ± SD from 8 values per group. (TIF) [file pone.0124881.s001.tif]
